# Decreased Expression of Cannabinoid Receptors in the Eutopic and Ectopic Endometrium of Patients with Adenomyosis

**DOI:** 10.1155/2019/5468954

**Published:** 2019-01-20

**Authors:** Xue Shen, Hua Duan, Sha Wang, Lu Gan, Qian Xu, Jin-Jiao Li

**Affiliations:** ^1^Department of Minimally Invasive Gynecologic Center, Beijing Obstetrics and Gynecology Hospital, Capital Medical University, Beijing 100006, China; ^2^Department of Obstetrics, Beijing Obstetrics and Gynecology Hospital, Capital Medical University, Beijing 100026, China

## Abstract

**Objective:**

Adenomyosis is a common gynecologic benign disease that may have a life-long negative impact on women. Previous studies have indicated that the endocannabinoid system may participate in the progress of endometriosis. Our research aims to analyze the expression patterns of the typical cannabinoid receptors (CB1 and CB2), the main constituents of the endocannabinoid system, in endometrial samples derived from patients diagnosed as adenomyosis or not.

**Methods:**

Eutopic and corresponding ectopic endometrium from 45 premenopausal women diagnosed as adenomyosis and normal endometrium from 34 age-matched women lacking evidence of adenomyosis were examined by immunohistochemistry and quantitative real-time polymerase chain reaction (qRT-PCR) to determine the CB1 and CB2 expression levels.

**Results:**

In either the proliferative or the secretory phase, CB1 and CB2 protein and mRNA levels were both significantly lower in the eutopic and ectopic endometrium of adenomyosis when compared with normal endometrium. For women with adenomyosis, CB1 and CB2 protein and mRNA levels were much lower in the ectopic endometrium than the eutopic in both phases of the cycle. Both CB1 and CB2 protein and mRNA levels were increased during the secretory phase in normal endometrium, while CB1 lost its cyclic variation in the eutopic and ectopic endometrium from patients diagnosed as adenomyosis.

**Conclusion:**

The decreased expression of CB1 and CB2 in the eutopic and ectopic endometrium from patients diagnosed as adenomyosis suggests that cannabinoid receptors may participate in the pathogenesis of adenomyosis.

## 1. Introduction

Adenomyosis is a familiar estrogen-dependent uterine disorder distinguished by a nonmalignant invasion of the bioactive endometrium into the myometrial wall, which may result in abnormal uterine bleeding, dysmenorrhea, and subfertility. This condition affects approximately 20% of women of reproductive age and shows an increased incidence among women with infertility [1, 2]. Despite the high prevalence and severe impact of this condition on women of various ages, little is known about its etiology and its pathogenesis [3]. Given that the uterus means a lot to women, medical therapy causes a big effect in the management of adenomyosis, and the rationale for it rests on the pathogenetic mechanisms of adenomyosis, which have much in common with endometriosis [4].

The endocannabinoid system (ECS) comprises a series of endogenously produced bioactive lipids (also known as endocannabinoids, eCBs), their specific eCB receptors, and the enzymes in charge of the synthesis, transport, and degradation of eCBs [5, 6]. The two major eCBs are anandamide (AEA) and 2-arachidonoylglycerol (2-AG), which are biosynthesized “on demand” from phospholipids and released instantly [7]. The enzymes involved in those processes are endocannabinoid metabolic enzymes, among which the N-acyl-phosphatidylethanolamine- (NAPE-) hydrolyzing phospholipase D (NAPE-PLD) and the fatty acid amide hydrolase (FAAH) are in charge of the biosynthesis and degradation of AEA, respectively; the* sn*-1 selective diacylglycerol lipase (DAGL) and the monoacylglycerol lipase (MAGL) are responsible for the formation and hydrolyzation of 2-AG, respectively [7, 8].

The eCBs are endogenous ligands for eCB receptors, and they have several effects mediated by the two classical receptors, the cannabinoid receptor 1 (CB1) and cannabinoid receptor 2 (CB2) [6]. CB1 is found to be sufficient in the central nervous system and is also reported to be highly expressed in the testis, uterus, ovaries, prostate, and placenta. In contrast, CB2 is mainly discovered in immune-based tissues and some neurons under certain pathologic conditions [9].

Although the primary focus on eCB biology has been in the fields of neurology and psychiatry, up to now numerous data have demonstrated the importance of the ECS and the therapeutic potential of cannabinoids in various diseases, such as immune diseases, endocrine and metabolic disorders, cardiovascular diseases, digestive and renal diseases and cancer [10, 11]. In particular, recent works have revealed certain interactions between the ECS and endometriosis, including alterations of components of the ECS, regulation of cell proliferation and apoptosis, cell invasion and migration, immunity or inflammation, innervation and pain perception [12, 13]. Furthermore, the ECS has been shown to affect angiogenesis in several tumors and fibrogenesis in fibrotic disease [14, 15]. Based on the above knowledge, the ECS could be a potential target to treat endometriosis [16]. However, there are limited data on the role of the ECS in the occurrence and development of adenomyosis.

Because adenomyosis has many similarities to endometriosis in definition, symptomology and pathogenetic mechanisms [17], we hypothesize that similar alterations of the ECS may also exist in adenomyosis. Using immunohistochemistry analysis and quantitative real-time polymerase chain reaction (qRT-PCR), we examined the expression patterns of CB1 and CB2 in the eutopic and ectopic endometrium from adenomyosis patients and compared them with CB1 and CB2 expression in the normal endometrium.

## 2. Materials and Methods

### 2.1. Sample Collection

Forty-five women diagnosed with adenomyosis and thirty-four women with no evidence of adenomyosis were recruited in this study. All of them had undergone a hysterectomy at Beijing Obstetrics and Gynecology Hospital from July 2016 to January 2018. Eutopic and ectopic endometrium from the adenomyosis group and normal endometrium from the control group were obtained during surgery and fixed in 10% formaldehyde for 24–48 h for the immunohistochemical assay. All of the patients were diagnosed with a postoperative pathological examination, and the endometrial dating was determined simultaneously as reported [18]. All patients had normal menstrual cycles (21–35 days), no evidence of endometriosis or endometrial pathology and malignancy, and no history of intrauterine device placement or hormone therapy within three months before the surgery. This study complied with the terms listed in the Declaration of Helsinki and was authorized by the ethics committee of our hospital (No. 2016-KY-012). Informed consent was signed by all patients before surgery for the use of their examination results and biological material.

### 2.2. Immunohistochemistry

From each sample, 4 *μ*m sections were prepared and dewaxed in xylene, rehydrated through graded alcohol and rinsed in distilled water. For antigen retrieval, they were boiled in citric saline (10 mmol/L, pH 6.0) for half an hour. Then, the samples were treated with 3% hydrogen peroxide solution for 25 min to block the activity of endogenous peroxidase. After the samples were blocked with 3% bovine serum albumin (BSA, Servicebio, Wuhan, China) for 30 min at room temperature, they were incubated with either a CB1 rabbit polyclonal antibody (C2866, Sigma, USA, dilution 1:300) or a CB2 rabbit polyclonal antibody (ab3561, Abcam, UK; dilution 1:100) at 4°C overnight. For negative controls, PBS was used instead. Next, the sections were rinsed in phosphate-buffered saline (PBS) 3 times and afterwards incubated with a horseradish peroxidase-labeled goat anti-rabbit antibody (Servicebio; dilution 1:200) for 50 min at room temperature. After washing the sections with PBS and incubating with 3,3-diaminobenzidine tetrahydrochloride dihydrate (Servicebio), they were counterstained with hematoxylin for 3 min. Finally, all slides were mounted with Permount (Servicebio) on glass slides, examined by a Leica DM4000B microscope (Leica, Wetzlar, Germany) and imaged with the Leica Application Suite (LAS, version 4.9.0, Leica). Immunohistochemical staining for CB1 and CB2 was assessed with the software Image-Pro-Plus 6.0 (Media Cybernetics, Rockville, Maryland) as reported previously [19] without knowing of patients' information. A series of 10 images of each section were randomly extracted for each targeted protein to get an average value for statistical comparison. Color intensity was used to define staining, and a color mask was performed. Then, we applied the mask equally to all images and acquired the measurements. The mean optical density (MOD), which means the ratio of the integrated optical density (IOD) to the total stained area of the endometrium, was recorded and herein equivalent to the immunoreactivity level of the target substance in the endometrium.

### 2.3. RNA Isolation and qRT-PCR

Total RNA of each sample was extracted with RNAiso Plus (Takara Bio Inc., Shiga, Japan) and quantified with a NanoDrop 2000/2000c Spectrophotometer (Thermo Fisher Scientific Inc., Massachusetts, USA). The PrimeScriptTMRT reagent Kit (RR047A, Takara) was used to synthesize cDNA from 1 *μ*g of total RNA per sample. The primers used in this study were designed by Sangon Biotech Co., Ltd. Shanghai, China. And the sequences are presented in Table 1. PCR reactions were performed on an AB 7500 Real-Time Polymerase Chain Reaction System (Applied Biosystems, Grand Island, New York) with the protocol for the SYBR® Premix Ex Taq™ II (RR820A, Takara). The reaction conditions were started at 95°C for 30 seconds as an initial denaturation, and then followed by 40 cycles of 5 seconds at 95°C and 34 seconds at 60°C. The 2^−△CT^ method was used to analyze the relative gene expression as reported [20], where △CT = CT (gene of target) – CT (internal control).

### 2.4. Statistical Analysis

The Statistical Programs for the Social Sciences software (SPSS, version 23.0 for Mac, Chicago, IL) was used to complete the all statistical analyses. Whether the values were distributed normally was assessed by the Shapiro-Wilk test. After that, we used the Mann-Whitney U test to evaluate the differences between the two menstrual phases among the normal, eutopic, and ectopic endometrium, and nonparametric analysis of variance on ranks (Kruskal-Wallis test) for pairwise multiple comparisons. Significance was arranged at a P value of < 0.05.

## 3. Results

We recruited 45 premenopausal women with adenomyosis as the adenomyosis group and 34 lacking evidence of adenomyosis as the control group. The mean ages of the two groups were 46.0 (standard deviation = 3.8, range = 37–53) years and 44.2 (standard deviation = 5.0, range = 32–53) years, respectively. There were 23 women in the proliferative phase and 22 in the secretory phase in the adenomyosis group, and in the control group, 22 in the proliferative phase and 12 in the secretory phase. No significant difference was observed in age and menstrual cycle between the two groups (*P* = 0.078 for Student's t-test, and *P* = 0.227 for Chi-Square tests, respectively).

### 3.1. CB1 and CB2 Protein Expression Levels in Normal, Eutopic, and Ectopic Endometrium

**Figure 1:**
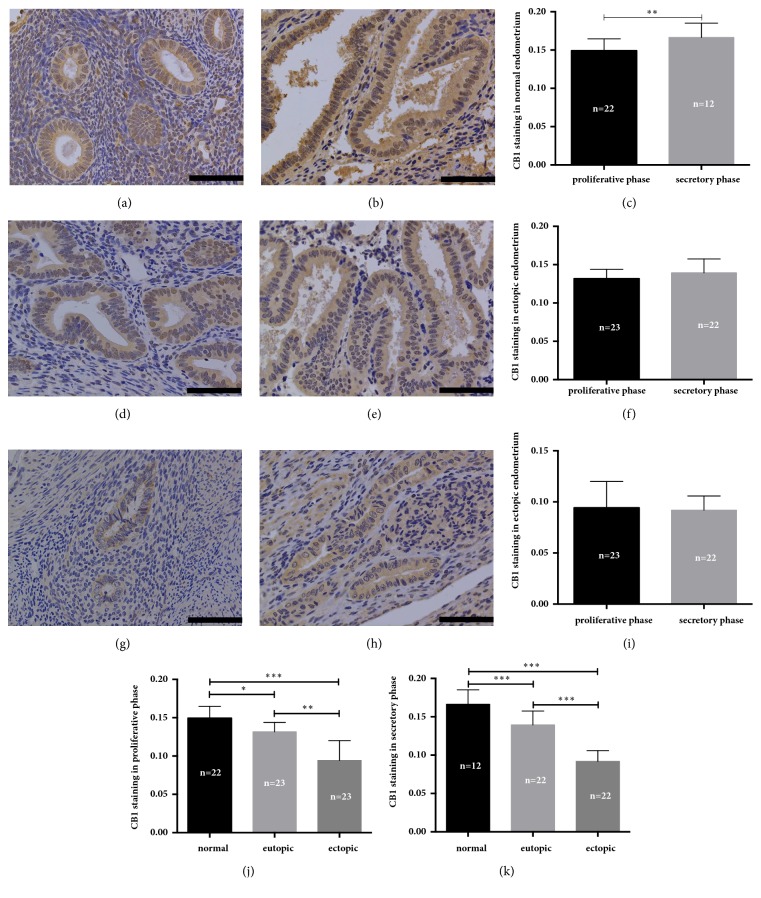
Immunohistochemical staining of CB1. (a) Normal endometrium in the proliferative phase. (b) Normal endometrium in the secretory phase. (c) Quantitative analysis of the mean optical density (MOD) between proliferative phase and secretory phase in normal endometrium. (d) Eutopic endometrium in the proliferative phase. (e) Eutopic endometrium in the secretory phase. (f) Quantitative analysis of MOD values between proliferative phase and secretory phase in eutopic endometrium. (g) Ectopic endometrium in the proliferative phase. (h) Ectopic endometrium in the secretory phase. (i) Quantitative analysis of MOD values between proliferative phase and secretory phase in ectopic endometrium. (j) and (k) Quantitative analysis of MOD values among normal endometrium, eutopic endometrium and ectopic endometrium in proliferative phase and secretory phase, respectively. All magnifications of the micrographs were × 400. Scale bars represent 5 *μ*m. The error bars on all histograms represent the standard deviation. *∗*, *P* < 0.05; *∗∗*, *P* < 0.01; *∗∗∗*, *P* < 0.001.

**Figure 2:**
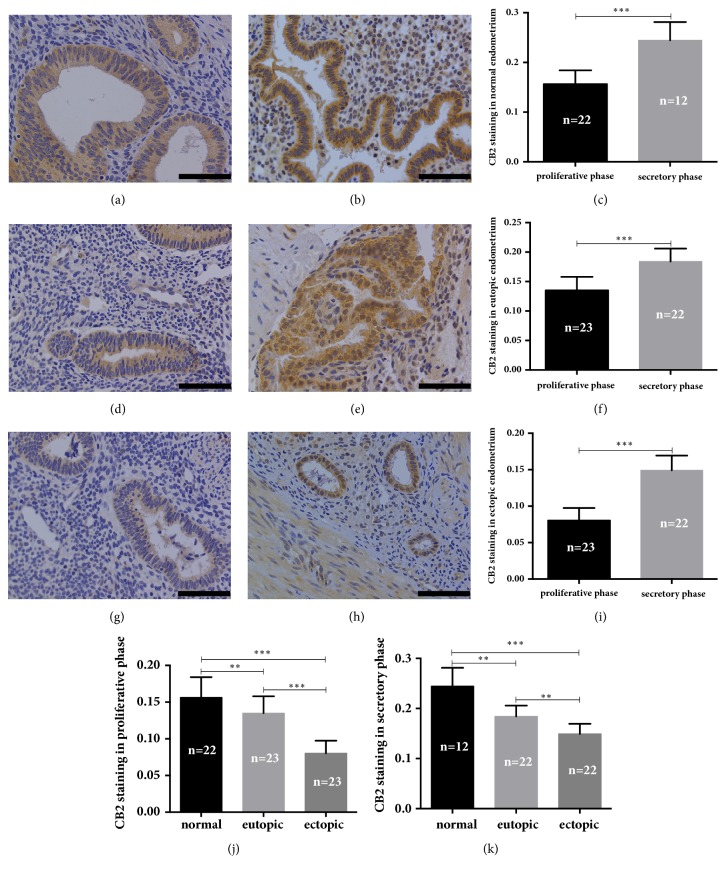
Immunohistochemical staining of CB2. (a) Normal endometrium in the proliferative phase. (b) Normal endometrium in the secretory phase. (c) Quantitative analysis of the mean optical density (MOD) between proliferative phase and secretory phase in normal endometrium. (d) Eutopic endometrium in the proliferative phase. (e) Eutopic endometrium in the secretory phase. (f) Quantitative analysis of MOD values between proliferative phase and secretory phase in eutopic endometrium. (g) Ectopic endometrium in the proliferative phase. (h) Ectopic endometrium in the secretory phase. (i) Quantitative analysis of MOD values between proliferative phase and secretory phase in ectopic endometrium. (j) and (k) Quantitative analysis of MOD values among normal endometrium, eutopic endometrium, and ectopic endometrium in proliferative phase and secretory phase, respectively. All magnifications of the micrographs were × 400. Scale bars represent 5 *μ*m. The error bars on all histograms represent the standard deviation. *∗*, *P* < 0.05; *∗∗*, *P* < 0.01; *∗∗∗*, *P* < 0.001.

The CB1 and CB2 receptors are both stained throughout the uterine tissue, not only in the endometrium but also in the myometrium and vascular smooth muscle cells. Immunoreactivity of both CB1 and CB2 was less intense in the stroma than in the glandular epithelium (Figures 1 and 2). The MOD of CB1 staining was decreased significantly in both the eutopic and ectopic endometrium from adenomyosis than in the normal endometrium (for the proliferative phase *P* = 0.028 and *P* < 0.001, respectively; for the secretory phase, both *P* < 0.001) (Figures 1(j) and 1(k)). The CB1 staining in the ectopic endometrium was less intense than that in the eutopic endometrium in the adenomyosis group (for the proliferative phase *P* = 0.001; for the secretory phase *P* < 0.001) (Figures 1(j) and 1(k)). In the normal endometrium, CB1 immunoreactivity in the secretory phase of the menstrual cycle was higher than that in the proliferative phase (*P* = 0.007) (Figure 1(c)). However, cyclic variation of CB1 immunoreactivity was not observed in either the eutopic endometrium or the ectopic endometrium (*P* = 0.098 and *P* = 0.991, respectively) (Figures 1(f) and 1(i)).

For CB2 staining, we observed similar patterns that it was less intense in both the eutopic and ectopic endometrium than in normal endometrium (for the proliferative phase *P* = 0.008 and *P* < 0.001, respectively; for the secretory phase, *P* = 0.009 and *P* < 0.001, respectively) (Figures 2(j) and 2(k)). When matched samples of the eutopic and ectopic endometrium of subjects diagnosed as adenomyosis were compared, the CB2 immunoreactivity was much lower in the ectopic endometrium than in the eutopic endometrium (for the proliferative phase *P* < 0.001; for the secretory phase *P* = 0.001) (Figures 2(j) and 2(k)). For each group, CB2 immunoreactivity was significantly higher in the secretory phase than in the proliferative phase (all *P* values < 0.001) (Figures 2(c), 2(f), and 2(i)).

### 3.2. CB1 and CB2 mRNA Expression Levels in Normal, Eutopic, and Ectopic Endometrium

To further determine the human endometrial CB1 and CB2 expression levels in patients with adenomyosis and controls, we observed CB1 and CB2 mRNA expression levels by qRT-PCR and obtained similar results. Both CB1 and CB2 mRNA levels were significantly lower in the eutopic and ectopic endometrium from patients with adenomyosis than the normal endometrium, and they were both significantly lower in the ectopic endometrium than in the matched eutopic endometrium (all *P* values < 0.05) (Table 2). In normal endometrium, both were significantly increased in the secretory phase (both *P* values < 0.05) (Table 2). However, in the eutopic and ectopic endometrium from adenomyosis uterus, CB1 lost its cyclic variation (*P* = 0.427 and *P* = 1, respectively), while CB2 retained its variation (both *P* values < 0.05) (Table 2).

## 4. Discussion

In this study, we found that the two classical cannabinoid receptors CB1 and CB2 were both reduced in the eutopic and ectopic endometrium from adenomyosis patients, regardless of the menstrual phase. When the eutopic endometrium and matched ectopic endometrium within the adenomyosis group were compared, CB1 and CB2 protein and mRNA levels were all lower in the ectopic endometrium in both phases of the cycle.

Limited data are available on the mechanism of the ECS in adenomyosis, but several studies have reported a relationship between this system and endometriosis. Our findings for CB1 expression are consistent with the report of Resuehr et al. [21], who demonstrated that CB1 expression was highest in the secretory phase of the control group and lowest in endometrial tissue of endometriosis, regardless of the menstrual cycle. However, these findings were different from the results of Leconte et al. [22], who reported that the CB1 and CB2 were expressed equally in the epithelial and stromal cell lines originated from eutopic endometrium and endometriosis, and other scholars [23] reported that no difference was found in endometrial CB1 immunoreactivity throughout the menstrual cycle. Evidence from studies on the ECS in reproduction has suggested that steroid hormones may affect the ECS [24, 25], plasma AEA is proved to fluctuate with the menstrual cycle and reach a peak at ovulation [26], and Di Blasio et al. [27] reviewed that the levels of FAAH, NAPE-PLD, CB1, and CB2 all change with sex hormones in female reproductive tissues. These are in accordance with the cyclic changes we observed in the expression of CB1 and CB2, and the cyclic changes of ECS have been considered vital in reproduction [28]. The conflicting results mentioned above may attribute to a moderate sample size with respect to the menstrual cycle in some studies; to methodological differences between different studies, such as immunohistochemistry used for human endometrial tissues [21, 23] and western blotting used on primary cultured endometriotic cells [22]; or to inappropriate comparison groups, such as the patients observed in study of Taylor et al. [23], whose complaints included menorrhagia and leiomyoma, which were not appropriate for comparison with endometriosis or adenomyosis.

Although some studies suggested that the myometrium plays a role in the progress of adenomyosis, the theories of the pathogenesis mainly originate in observations on cyclical endometrial changes and their function [29]. At present, adenomyosis is generally considered as an estrogen-dependent disease represented with increased inflammation, fibrosis, neuroangiogenesis, and abnormal uterine contractility [17]. The cannabinoid receptors have long been known to have a role in inflammatory regulation [30–32]. Iuvone et al. [33] showed that cannabinoid receptors were present in inflammatory endometrial tissue, and selective activation of CB2 is associated with the nitric oxide release process existing in endometrial inflammation. Additionally, many studies found that the mutual effect between the ECS and immune system may affect the modulation of biological processes contained in implantation [34–38]. Extensive data have shown that by regulating the production of cytokine, chemotaxis, and proliferation, the ECS has participated in regulating several immune cell lineages found to be critical in the maintenance of normal pregnancy [39]. Based on the current knowledge and our results, we hypothesized that the decreased expression of CB1 and CB2 observed in endometrial tissues of adenomyosis may influence inflammatory changes as well as adenomyosis-associated infertility.

Furthermore, animal studies have demonstrated that up-regulation of CB1 and CB2 may selectively inhibit myometrial spontaneous contractility, and if a similar mechanism exists in the human uterus, hypercontractility present in adenomyosis patients may be alleviated through activation of these two receptors [40, 41]. Concerning the other characteristics of adenomyosis mentioned above, an antifibrotic role has been shown in a variety of fibrotic diseases by targeting the cannabinoid receptor CB1 or CB2 [42–46]. Cannabinoid receptors have also been demonstrated to participate in tumor angiogenesis and invasiveness [15, 47, 48]. In addition, CB1 was found to promote innervation and the growth of ectopic lesions in animal models of endometriosis [12, 49]. Given that adenomyosis is closely related to endometriosis and the evidence above, we hypothesized that the aberrant expression of CB1 and CB2 might participate in the pathogenesis of adenomyosis.

Other interesting results from our study were the differential expression levels of CB1 and CB2 between the eutopic and ectopic endometrium in adenomyosis, and the finding that CB1 lost its cyclic variation in endometrial tissue of adenomyosis. With regard to the differential expression between the eutopic and ectopic endometrium, numerous publications have been reviewed by Yen et al. [29]. Maybe this difference between them further supports the hypothesis of “metaplasia” rather than “invagination” for the pathogenesis of adenomyosis [3]. The lack of cyclic variation in CB1 in adenomyosis suggests that it plays a more important role than CB2 in the pathogenesis of adenomyosis, which appears consistent with the evidence collected by Maccarrone et al. [50] showing that CB1 is more important in female fertility than CB2.

This study is based on a fairly large sample size of patients, especially when considering the impact of the menstrual cycle. As a preliminary observational study, this report has several possible limitations. First, the serous expression of eCBs, which may have an impact on the levels of CB1 and CB2 were not detected. Second, we did not perform further functional investigations. Therefore, the next steps would be additional investigations on the association of these two cannabinoid receptors with the clinical characteristics of adenomyosis and potential cytokines, immune cells, and molecular pathways.

## 5. Conclusions

In conclusion, we found a significant decrease in the cannabinoid receptors CB1 and CB2 in the eutopic and ectopic endometrium of patients with adenomyosis, regardless of the menstrual phase, suggesting that CB1 and CB2 participate in the pathogenesis of this condition. Further studies on the role of the ECS in adenomyosis will be helpful to determine whether this system could be a novel target to treat adenomyosis.

## Figures and Tables

**Table 1:** Primers of specific genes used in qRT-PCR analyses.

Gene		Sequence (5′-3′)
CB1	Forward	5′-CCTAGATGGCCTTGCAGATACC-3′
	Reverse	5′- GAATGTCATTTGAGCCCACGTA-3′
CB2	Forward	5′-CAGGTCAAGAAGGCCTTTGC-3′
	Reverse	5′-GCATAGATGACAGGGTTGACCAT-3′
*β*-actin	Forward	5′-TGCCGACAGGATGCAGAAG-3′
	Reverse	5′-CTCAGGAGGAGCAATGATCTTGA-3′

**Table 2:** Relative expression levels of CB1 mRNA and CB2 mRNA.

	normal	eutopic	ectopic
	(n = 34)	(n = 45)	(n = 45)
CB1 mRNA levels			
proliferative phase	0.153 ± 0.028^a,c,d^	0.071 ± 0.018^a,b,e^	0.022 ± 0.006^b,c,e^
	(n = 22)	(n = 23)	(n = 23)
secretory phase	0.244 ± 0.038^a,c,d^	0.127 ± 0.041^a,b,e^	0.027 ± 0.007^b,c,e^
	(n = 12)	(n = 22)	(n = 22)
CB2 mRNA levels			
proliferative phase	0.183 ± 0.026^a,c,d^	0.041 ± 0.006^a,b,d^	0.015 ± 0.005^b,c,d^
	(n = 22)	(n = 23)	(n = 23)
secretory phase	0.313 ± 0.053^a,c,d^	0.148 ± 0.032^a,b,d^	0.029 ± 0.006^b,c,d^
	(n = 12)	(n = 22)	(n = 22)

Values given as mean ± standard error (SEM). ^a^Comparison between normal endometrium and eutopic endometrium (same phase), *p* < 0.05. ^b^Comparison between eutopic endometrium and ectopic endometrium (same phase), *p* < 0.05. ^c^Comparison between normal endometrium and ectopic endometrium (same phase), *p* < 0.05. ^d^Comparison between proliferative phase and secretory phase (same group), *p* < 0.05. ^e^Comparison between proliferative phase and secretory phase (same group), *p* ≧ 0.05.

## Data Availability

The data used to support the findings of this study are available from the corresponding author upon request.
